# Media narratives about organ and tissue donation and transplantation: reflective analysis of published content

**DOI:** 10.1590/1980-220X-REEUSP-2024-0342en

**Published:** 2025-12-01

**Authors:** Gleyciane Santos Vieira, Rafael Rodrigo da Silva Pimentel, Rosane Almeida de Freitas, Marcelo José dos Santos

**Affiliations:** 1 Universidade de São Paulo, Escola de Enfermagem, São Paulo, SP, Brazil.; 2 Hospital Israelita Albert Einstein, Centro de Estudos, Pesquisa e Prática em Atenção Primária à Saúde e Redes, São Paulo, SP, Brazil.; 3 Universidade Estadual de Maringá, Maringá, PR, Brazil.

**Keywords:** Electronic Publications, Tissue and Organ Procurement, News

## Abstract

**Objective:**

To analyze the frequency and content of publications on organ and tissue donation and transplantation in the digital newspapers “*Folha de São Paulo*” and “*O Estado de São Paulo*” between 2018 and 2023.

**Method:**

Through a quantitative-qualitative approach, 317 reports were examined based on thematic and discursive criteria, with the aid of SPSS^®^ and IRAMUTEQ^®^ software.

**Results:**

The data revealed a predominance of positive content (71.9%), concentrated on the topic “transplant” (60.57%), and in 2023, with peaks in August and September. The main categories were “incentive/promotion” and “information”, with the heart being the most cited organ. Textual analysis highlighted topics such as transplantation, family refusal, xenotransplantation, and the impact of the pandemic.

**Conclusion:**

Digital media exerts significant influence on public perception of donation and transplantation. However, there is isolated emphasis on certain stages of the process, which can compromise overall understanding. Continuous and integrated communication strategies are essential to strengthen the donation culture.

## INTRODUCTION

Organ and tissue donation represents an act of social relevance capable of saving or improving the quality of life by offering hope to thousands of patients on the waiting list^(1)^. In Brazil, the demand for transplants remains growing, with around 59,000 people waiting for an organ or tissue in 2023^(2)^. Despite progress, persistent challenges, such as family refusal, lack of knowledge about brain death criteria, and the shortage of effective donors, limit the reach of this process, highlighting the need for strategies to increase public awareness^(3,4,5)^.

In this context, the media, through soap operas, series, films, advertising campaigns, magazines, and digital newspapers, plays a prominent role as an informational and promotional tool on health-related issues, as it provides an easily accessible and low-cost means of obtaining references. The media, therefore, becomes the holder of knowledge and can directly or indirectly impact the community’s attitudes and decision-making, depending on the content and structure of its studies, positively or negatively influencing the organ and tissue donation process^(6,7,8,9)^. However, the impact of the media is ambivalent: sensationalist or distorted content can reinforce myths, damaging society’s trust^(8)^.

Publications about celebrity organ donation, such as the case of Brazilian host Augusto Liberato, who donated his organs and tissues after a domestic accident that caused his brain death in November 2019 in Florida, United States of America, had a large impact, being associated with the increase in the number of donations by the Brazilian Transplant Registry^(10)^. On the other hand, news with distorted and appealing information about organ trafficking and erroneous diagnoses undermine the importance of organ and tissue donation^(8)^.

Although the media plays an important role in health issues, most available studies assess the impact of media on advertising campaigns on smoking, alcoholism, cancer, HIV/AIDS, among others^(6,9)^. There is a scarcity of national research specifically analyzing the role of media publications in the organ and tissue donation process. Most studies are limited to institutional data, such as those from the Brazilian Organ Transplant Association and the Ministry of Health, which are not readily available to the general public. This gap makes it important to investigate how media outlets with the widest reach—such as digital newspapers—approach the topic, as they are primary sources of information for the majority of the population.

Therefore, this study aimed to analyze the frequency and content of publications on organ donation and transplantation in two widely circulated digital newspapers in Brazil between 2018 and 2023.

## METHOD

### Study Design

This is a quantitative and qualitative documentary study on the content of reports and news published in two digital newspapers with national circulation, namely “*O Estado de São Paulo*”, known as “*Estadão*”, and “*Folha de São Paulo*”.

### Study Location and Population

The study was conducted in two widely circulated digital newspapers in Brazil. The newspaper “*O Estado de São Paulo*” was founded in 1875 under the name “*A Província de São Paulo*”, when Brazil was still under the Monarchy of Dom Pedro II. In 1889, with the Proclamation of the Republic, the name was changed to “*O Estado de São Paulo*”. The newspaper has been available online since 1995.

The newspaper “*Folha de São Paulo*” was founded in 1960 after the merger of three dailies: “*Folha da Manhã*”, “*Folha da Noite*”, and “*Folha da Tarde*”. In 1995, the first online news site, “*FolhaWeb*”, was launched.

### Selection Criteria

All news stories that addressed the topic in both the paid and free versions were included. Four stories were excluded because they addressed the donation of cadavers and body parts for scientific purposes and personal issues that overlapped with the topic.

### Data Collection

Data collection took place from September 2023 to January 2024. In the first stage, news and reports were collected using filters with keywords (organ donation; organ and tissue donation; tissue donation; organ donor; tissue donor; living donor(s); deceased donor(s); organ transplant; organ and tissue transplant; tissue transplant; transplanted; organ transplanted; tissue transplanted; organ trafficking; family refusal of donation; family refusal; brain death; brain death; and donation) published from January 2018 to December 2023. In this phase, articles were selected based on their title and abstract.

The second stage involved a thorough reading of the news and its organization in Microsoft Excel® and Microsoft Word® programs to deepen and broaden the visualization of publications’ contents.

### Data Analysis and Treatment

In quantitative analysis, data were extracted from a Microsoft Excel® spreadsheet and imported into the Statistical Package for the Social Sciences version 20.0 for analysis of simple (n) and relative (%) frequencies. To this end, publications’ contents were grouped according to their similar meaning in Microsoft Excel® and subsequently classified into categories, guided by Bardin’s thematic-categorical content analysis framework, which comprises the phases of pre-analysis, material exploration, and data treatment, inference, and interpretation^(11)^.

To conceptualize the media category, positive media was used for news that promoted and praised the topic; negative media was used for news that depreciated, problematized and criticized the topic^(12)^; and neutral media was used for those that did not express a preponderant bias. Each code for the categories was defined by skimming, according to the reference. Afterward, the code was defined according to the news content, and then peer reviews were conducted to confirm the coding for the construction of each category/class. Disagreements were resolved in group consensus rounds.

From the analysis, the codes that generated the categories emerged: media (positive, negative or neutral); news topic (donation, transplant or transplant/donation); organs/tissues and news class (incentive/promotion, information, impact of the COVID-19 pandemic, innovation, xenotransplantation, misinformation about donation/transplantation, legislation, waiting list, complications in the donation/transplantation process, breach of confidentiality of donors’ identity, family refusal, and transportation of organs and tissues).

Furthermore, qualitative analysis used the *Interface de R pour les Analyses Multidimensionalnelles de Textes et de Questionnaires* (IRAMUTEQ) version 0.7 alpha 2 and R version 3.2.3. Thus, the content of the news and reports (text *corpus*) was transcribed into Microsoft Word^®^ and revised to conform to the software’s specifications, such as the introduction of command lines, removal of paragraphs, correction of punctuation, standardization of acronyms, and grouping of significant words with underscores. To facilitate analysis, IRAMUTEQ divides the texts in the *corpus* into text segments (TS), generally three lines long^(13)^.

For this research, the following were used: 1) Word cloud, a visual representation of the frequency of terms in the *corpus* according to their size; 2) Similarity tree, which is based on graph theory, which indicates the connections between words; and 3) Dendrogram and factorial presentation, created through the Descending Hierarchical Classification (DHC), which groups words into classes in order to demonstrate their connection with each other through the chi-square test (*χ* ^2^).

The interpretation of classes resulting from DHC followed theoretical criteria based on content analysis^(11)^, combined with the perspective of discursive textual analysis^(14)^. Each class was examined based on the most characteristic words (with the highest chi-square value), the content of associated TSs, and their relationship to the full *corpus*. The assignment of meaning to the classes considered both the frequency and association of terms and emerging semantic contexts, enabling an interpretation that goes beyond lexical co-occurrence, anchored in the literature of the research thematic area. This approach is based on constructivist epistemological assumptions, understanding that the meaning of discourses is socially constructed and can be accessed through shared lexical structures. The classical textual analysis (DHC) used by IRAMUTEQ seeks to identify regularities and patterns in discourses, allowing the researcher to interpret latent meanings while preserving coherence with the research’s exploratory and qualitative objectives^(15)^.

### Ethical Aspects

The data collected in this research were taken from publicly available documentary sources. According to Resolution 510/2016 of the Brazilian National Health Council, there is no need for the project to be assessed by the Research Ethics Committee.

## RESULTS

The database consisted of 317 news items and reports, 185 (58.36%) of which were published by “*Folha de São Paulo*” and 132 (41.64%) by “*O Estado de São Paulo*”. The publications occurred between 2018 and 2023, with a significant number in 2023, with 159 news items (50.16%), while there was a smaller number in 2020, with only 20 reports (6.31%). When examining the distribution of news items in relation to the months of their publication, August and September stand out from the other months. Concerning the content of news items, 228 (71.9%) were considered positive and 54 (17.03%) were negative. The topics were mainly associated with transplantation, with 192 (60.57%), followed by donation, with 81 (25.55%) of publications (Table 1).

**Table 1:** Characterization of the news published in the newspapers “O Estado de São Paulo” and “Folha de São Paulo” (2018–2023) – São Paulo, SP, Brazil, 2024.

Variables	N (%)
**Newspaper**	
Folha de São Paulo	185 (58,36)
O Estado de São Paulo	132 (41,64)
**Month**	
January	16 (5,05)
February	8 (2,52)
March	19 (5,99)
April	9 (2,84)
May	16 (5,05)
June	14 (4,42)
July	16 (5,05)
August	85 (26,81)
September	60 (18,93)
October	25 (7,89)
November	31 (9,78)
December	18 (5,68)
**Year**	
2018	34 (10,73)
2019	26 (8,20)
2020	20 (6,31)
2021	34 (10,73)
2022	44 (13,88)
2023	159 (50,16)
**Media**	
Negative	54 (17,03)
Neutral	35 (11,04)
Positive	228 (71,92)
**Topic**	
Transplantation	192 (60,57)
Donation	81 (25,55)
Donation/transplantation	44 (13,88)

To improve the news characterization and quantification, they were divided into 12 classes, in which the incentive/promotion class was predominant, with 107 (33.75%) of publications, and, in last place, with only four news items (1.27%), the transportation class. Furthermore, in 145 news items (45.74%), organs and/or tissues were not specifically mentioned, followed by the heart, with 95 news items (29.97%) (Table 2).

**Table 2:** Classification of news published in the newspapers “O Estado de São Paulo” and “Folha de São Paulo” according to content and organs and tissues donated/transplanted and cited – São Paulo, SP, Brazil, 2024.

Variables	N (%)
**Detailed organs/tissues**	
Unspecified	145 (45,74)
Heart	95 (29,97)
Kidneys	37 (11,67)
Liver	12 (3,79)
Lung	8 (2,52)
Pancreas	7 (2,21)
Skin	4 (1,26)
Uterus	4 (1,26)
Intestine	2 (0,63)
Corneas	2 (0,63)
Bones	1 (0,32)
**Classes**	
Incentive/promotion	107 (33,75)
Information	65 (20,51)
Impact of the COVID-19 pandemic	28 (8,83)
Innovation	28 (8,83)
Xenotransplantation	25 (7,89)
Misinformation about donation/transplantation	15 (4,73)
Legislation	12 (3,79)
Waiting list	10 (3,15)
Complications in the donation/transplant process	8 (3,52)
Breach of donor confidentiality	8 (3,52)
Family refusal	7 (2,21)
Organ and tissue transportation	4 (1,27)

**Figure 1:**
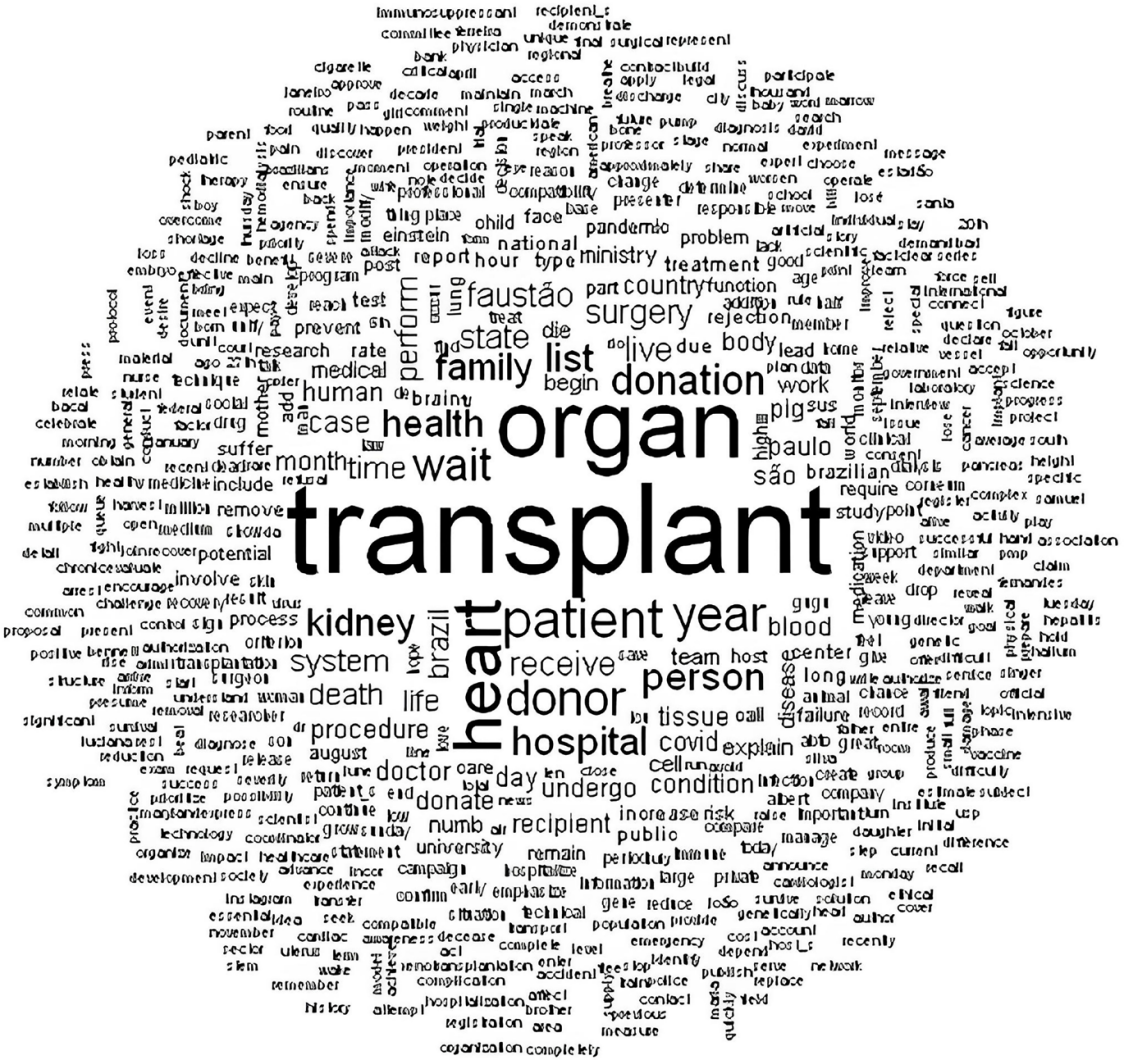
Word cloud identified in reports from the newspapers “*O Estado de São Paulo*” and “*Folha de São Paulo*” on organ and tissue donation and transplantation – São Paulo, SP, Brazil, 2024.

In qualitative analysis, the words “patient”, “year”, “transplant”, “say”, “organ”, “physician”, “heart”, among others, appear highlighted in the word cloud (Figure 1). In contrast, other words appear less frequently, such as “receive”, “surgery”, “donor”, and “procedure”.

**Figure 2:**
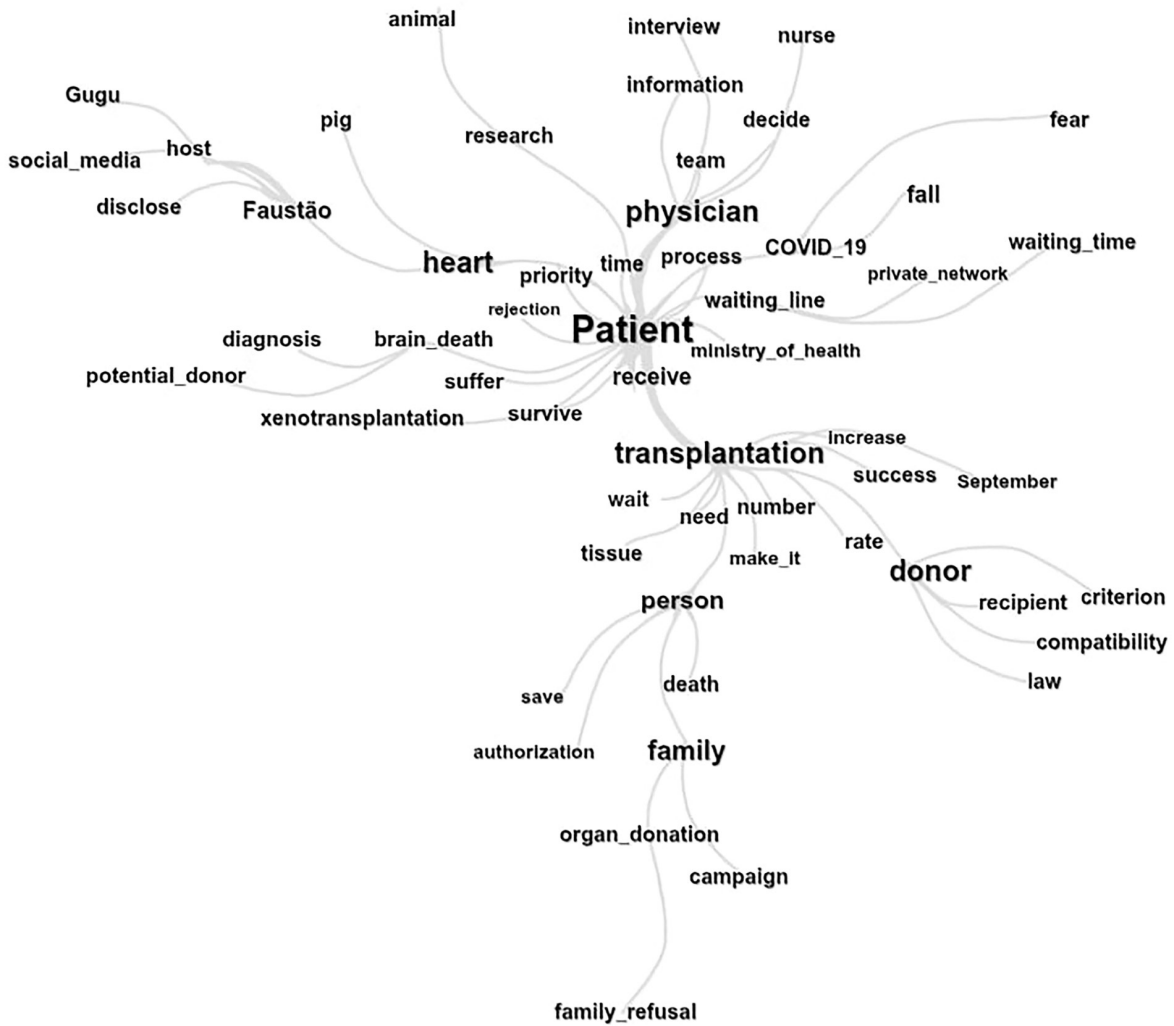
Word similarity tree found in texts published in the newspapers “*O Estado de São Paulo*” and “*Folha de São Paulo*” regarding organ and tissue donation and transplantation – São Paulo, SP, Brazil, 2024.

It can be related to the similarity tree (Figure 2), which shows the relationship between the TS “patient” and the “waiting list” of individuals (“people”) waiting for (“receiving”) an organ or tissue for transplant. The figure of a “physician” refers to the professionals who are part of the team involved in the organ and tissue donation (“donor”) and (“transplantation”) process, and the “heart”.

**Figure 3:**
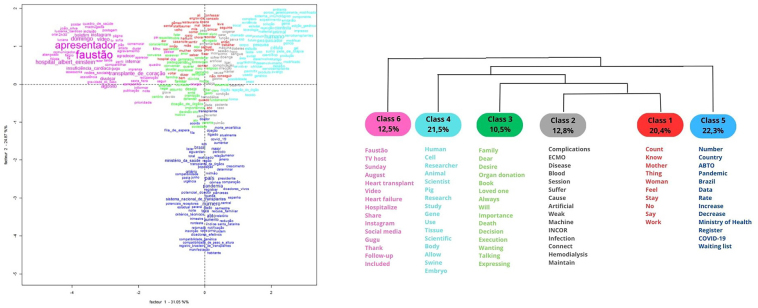
Factorial presentation and dendrogram of the Descending Hierarchical Classification, found in texts published in the newspapers “*O Estado de São Paulo*” and “*Folha de São Paulo*”, referring to organ and tissue donation and transplantation – São Paulo, SP, Brazil, 2024.

In the factorial and DHC presentation (Figure 3), the content generated six classes, with 20.4%, 12.8%, 10.5%, 21.5%, 22.3%, and 12.5% of TSs, respectively. Class 1 addresses the importance of individuals informing their families of their wishes regarding donation and the decision they made based on this; class 2 highlights the health complications that are used as priority criteria for organ and tissue transplantation; class 3 discusses the importance of the family’s decision to respect the wish to donate organs and tissues; class 4 refers to studies to use pig organs in humans (xenotransplantation) as a resource to reduce the shortage of organs for transplantation and, thus, reduce the waiting list; class 5 relates the impacts of COVID-19 on transplant rates in Brazil; and class 6 refers to issues related to the dissemination of cases on social media, such as heart transplantation of host Faustão and organ donation by host Gugu.

## DISCUSSION

The analysis highlighted the key role of digital media in promoting organ donation and transplantation, especially through positive, testimonial-based content capable of mobilizing emotions and broadening public debate on the topic. The literature supports this finding by demonstrating that transformational narratives tend to be more effective than purely informative ones in generating social engagement^(6,16,17)^. This effect is enhanced by indirect strategies, such as the intensification of publications on commemorative dates and the predominance of positive tones in articles, which contributes to a more welcoming perception of the donation process^(6,16)^. A study published in 2023 analyzed the content of 313 English-language posts about organ donation on the social media platform TikTok^®^ and found that a significant number of videos promoted organ donation and combating misinformation, with a predominance of positive content^(12)^. Thus, it can be stated that the availability of organs for transplantation is influenced by social judgment, which can be modified with appropriate stimuli^(7)^.

High-profile cases, such as those involving Brazilian TV hosts Fausto Silva (Faustão) and Augusto Liberato (Gugu), serve as triggers for an increase in publications. While they serve as an opportunity to clarify how the transplant system works and combat misinformation, these reports also reveal the influence of external factors such as fame and social status on the media visibility of the issue, which generates ambiguous perceptions about justice and access to the system^(18)^. The press’s response to these situations demonstrates significant educational potential, but reinforces the need for clearer guidelines on how to responsibly approach these narratives.

Therefore, the predominance of the topic “transplantation” over “donation” and the underrepresentation of specific organs and tissues, such as corneas, bones, and skin, reveal an asymmetry in news coverage. This imbalance can compromise public understanding of the interdependence between these stages, as already highlighted in other investigations^(5,19,20)^. By prioritizing recipients and their recovery — often associated with feelings of overcoming and gratitude — over the donation process and the figure of the donor, the media contributes to a partial narrative, which silences the complexity and emotional burden involved in the family decision to donate^(21)^.

Another critical point is the fragmented treatment of the process stages, which may arise from the editorial structure of the media. While understandable from a journalistic perspective, this approach risks hindering the perception of donation and transplantation as an integrated cycle, compromising informed public engagement. This fragmentation also contributes to the invisibility of relevant topics, such as family refusal, brain death, and tissue donation—issues that require not only technical information but also cultural and ethical sensitivity^(4,5,22,23)^.

The COVID-19 pandemic reinforced this unstable scenario by negatively impacting donation and transplant rates for clinical, logistical, and biosafety reasons^(24,25)^. Still, the expanded use of telemedicine to monitor recipients demonstrates the system’s adaptive capacity, suggesting possibilities for innovation in the future.

In this context, the advancement of research on xenotransplantation is also noteworthy as a response to the shortage of donors, with national and international initiatives that reinforce the urgency of complementary solutions without, however, replacing the importance of human donation^(26,27,28)^.

The evidence from this research indicates that strengthening the donation culture requires a coordinated effort between the press, healthcare professionals, and public officials. The creation of guidelines, such as those developed by international institutions^(18)^, can inspire national initiatives aimed at improving communication practices, combating sensationalism, and promoting ethical, clear, and evidence-based information. These findings point to the need for intersectoral coordination between the media, healthcare services, and public institutions, with a view to developing continuous and responsible communication strategies for organ and tissue donation and transplantation. The development of support materials—such as guides for journalists—that address ethical, technical, and cultural aspects is recommended, promoting sensitive, educational, and evidence-based coverage.

This study’s limitation is that its analysis is restricted to two nationally circulated digital newspapers, which may not represent the diversity of approaches adopted by regional, alternative media outlets, or other digital platforms. Furthermore, timeframe and use of specific keywords may have excluded content relevant to the topic. Finally, by prioritizing the analysis of published discourse, it was not possible to capture the effects of how these messages were received by the public, which limits inferences about their direct social impact.

From a research perspective, future investigations could expand the analysis to other media outlets and social networks as well as explore how messages are received by different audiences. Qualitative studies with family members of potential donors could also deepen the understanding of how media messages impact the decision to donate. Furthermore, it is recommended to examine communication strategies for tissue donation, whose visibility remains limited.

## CONCLUSION

Based on the above, it is clear that, although online newspapers analyze the topic of organ and tissue donation and transplantation from a positive perspective, the approach remains fragmented, with an emphasis on transplantation over donation. This imbalance can limit public understanding of the interdependence between these stages and compromise public engagement with the cause.

The results demonstrate that high-profile events, such as cases involving public figures, directly influence the volume and tone of studies, indicating that the media responds to temporary factors rather than adopting a continuous information strategy. This reinforces the need for stronger collaboration between healthcare professionals, journalists, and public officials to promote ethical and educational coverage, aiming to reduce misinformation and combat stigma. Investing in communication strategies is essential to strengthen the donation culture in Brazil and reduce the gap between information disseminated and public decision-making.

## Data Availability

The research data are available upon request.

## References

[1] Vanholder R, Domínguez-Gil B, Busic M, Cortez-Pinto H, Craig JC, Jager KJ (2021). Organ donation and transplantation: a multi-stakeholder call to action. Nat Rev Nephrol.

[2] Registro Brasileiro de Transplantes (2023). Dimensionamento dos Transplantes no Brasil e em cada estado [Internet].

[3] Araujo AT, Araujo HV, Cruz SRF, Souza RZ (2023). Os principais fatores de recusa de doação de órgãos e tecidos no âmbito familiar: revisão de literatura. Braz J Implantol Health Sci.

[4] Roza BA, Schuantes-Paim SM, Oliveira PC, Malosti RD, Knhis NDS, Menjivar A (2024). Reasons for organ and tissue donation refusal and opposition: a scoping review. Rev Panam Salud Publica.

[5] Pompeu MH, Silva SS, Roza BDA, Bueno SMV (2014). Fatores envolvidos na negativa da doação de tecido ósseo. Acta Paul Enferm.

[6] Wakefield MA, Loken B, Hornik RC (2010). Use of mass media campaigns to change health behaviour. Lancet.

[7] Feldens TK, Jacinto PA (2020). In: XXV ANPEC Nordeste; 2020 Oct 28-29 [Internet].

[8] Aykas A, Uslu A, Simsek C (2015). Mass media, online social network, and organ donation: old mistakes and new perspectives. Transplant Proc.

[9] Jiang X, Jiang W, Cai J, Su Q, Zhou Z, He L (2019). Characterizing media content and effects of organ donation on a social media platform: content analysis. J Med Internet Res.

[10] Registro Brasileiro de Transplantes (2019). Dimensionamento dos Transplantes no Brasil e em cada estado [Internet].

[11] Bardin L (2016). Análise de conteúdo.

[12] Marcon AR, Zenone M, Caulfield T (2023). The portrayal of organ donation on TikTok: a content analysis of popular English-language TikTok videos. Digit Health.

[13] Martins KM, Paula MC, Gomes LPS, Santos JE (2022). O software IRAMUTEQ como recurso para a análise textual discursiva. Rev Pesq Qual.

[14] Magno CMV, Gonçalves TVO (2023). O testemunho em pesquisa narrativa e a análise textual discursiva associada ao IRAMUTEQ. Amaz Rev Educ Ciênc Matemát.

[15] Camargo BV, Justo AM (2021). Tutorial para uso do software IRAMUTEQ (Interface de R pour les Analyses Multidimensionnelles de Textes et de Questionnaires) [Internet].

[16] Olsacher A, Bade C, Ehlers J, Fehring L (2023). How to effectively communicate health information on social media depending on the audience’s personality traits: an experimental study in the context of organ donation in Germany. Soc Sci Med.

[17] Rodrigue JR, Boger M, DuBay D, Fleishman A (2019). Increasing organ donor designation rates in adolescents: a cluster randomized trial. Am J Public Health.

[18] Organização Panamericana de la Salud (2021). Recomendações aos meios de comunicação sobre a abordagem responsável pelas notícias vinculadas à doação e ao transplante [Internet].

[19] Marcon AR, Caulfield T (2022). Donation and transplantation coverage in the Canadian media: a content analysis of story focus over 2 decades. Can J Surg.

[20] Harel I, Kogut T, Pinchas M, Slovic P (2017). Effect of media presentations on willingness to commit organ donation. Proc Natl Acad Sci USA.

[21] Santos JIR, Santos ADB, Lira GG, Moura LTR (2019). Percepções de familiares sobre a doação de órgãos e tecidos. Rev. Enferm UFPE.

[22] Moraes EL, Silva LBB, Santos MJ, Lima EAA, Massarollo MCKB (2015). Obstáculos no processo de doação de órgãos e estratégias para otimizar as taxas de consentimento familiar. Rev Bras Med.

[23] Brito ÁN, Santos MJD, Pimentel RRS (2024). Skin donation for transplantation: social representations of family members who (do not) give consent for collection. Burns.

[24] Garcia VD, Pêgo-Fernandes PM (2021). Transplante de órgãos e COVID-19. Sao Paulo Med J.

[25] Domínguez-Gil B, Coll E, Fernández-Ruiz M, Corral E, Del Río F, Zaragoza R (2020). COVID-19 in Spain: transplantation in the midst of the pandemic. Am J Transplant.

[26] Raia SMA (2022). Xenotransplante: uma perspectiva consistente. Rev Col Bras Cir.

[27] Galvão FHF, Carneiro D’Albuquerque LA (2020). Xenotransplante. Rev Med.

[28] Griffith BP, Goerlich CE, Singh AK, Rothblatt M, Lau CL, Shah A (2022). Genetically modified porcine-to-human cardiac xenotransplantation. N Engl J Med.

