# Supplementary material for: Media narratives about organ and tissue donation and transplantation: reflective analysis of published content
Source: Rev Esc Enferm USP. 2025 Dec 1;59:e20240342. doi: 10.1590/1980-220X-REEUSP-2024-0342en (PMC12680134; doi:10.1590/1980-220X-REEUSP-2024-0342en)
Supplement: Translation

# Translation: Narrativas midiáticas sobre doação e transplante de órgãos e tecidos: análise reflexiva do conteúdo publicado

**DOI:** 10.1590/1980-220X-REEUSP-2024-0342pt

**Authors:** Gleyciane Santos Vieira, Rafael Rodrigo da Silva Pimentel, Rosane Almeida de Freitas, Marcelo José dos Santos

**Affiliations:** 1 Universidade de São Paulo, Escola de Enfermagem, São Paulo, SP, Brasil.; 2 Hospital Israelita Albert Einstein, Centro de Estudos, Pesquisa e Prática em Atenção Primária à Saúde e Redes, São Paulo, SP, Brasil.; 3 Universidade Estadual de Maringá, Maringá, PR, Brasil.

**Keywords:** Publicações Eletrônicas, Obtenção de Tecidos e Órgãos, Notícias

## Abstract

**Objetivo:**

Analisar a frequência e o conteúdo das publicações sobre doação e transplante de órgãos e tecidos nos jornais digitais “Folha de São Paulo” e “O Estado de São Paulo” entre 2018 e 2023.

**Método:**

Por meio de uma abordagem quanti-qualitativa, 317 reportagens foram examinadas a partir de critérios temáticos e discursivos, com auxílio dos *softwares* SPSS^®^ e IRAMUTEQ^®^.

**Resultados:**

Os dados revelaram predominância de conteúdos com conteúdo positivo (71,9%) concentrados no tema “transplante” (60,57%) e, no ano de 2023, com picos em agosto e setembro. As principais categorias foram “incentivo/promoção” e “informação”, com destaque para o coração como órgão mais citado. A análise textual destacou temas como transplante, recusa familiar, xenotransplante e impacto da pandemia.

**Conclusão:**

A mídia digital exerce influência relevante na percepção pública sobre doação e transplante. No entanto, há ênfase isolada em etapas do processo, o que pode comprometer a compreensão global. Estratégias comunicacionais contínuas e integradas são essenciais para fortalecer a cultura da doação.

## INTRODUÇÃO

A doação de órgãos e tecidos representa um ato de relevância social capaz de salvar ou melhorar a qualidade de vida ao oferecer esperança a milhares de pacientes em lista de espera^(1)^. No Brasil, a demanda por transplantes permanece crescente, com cerca de 59 mil pessoas aguardando por um órgão ou tecido em 2023^(2)^. Apesar dos avanços, desafios persistentes, como a recusa familiar, a falta de conhecimento sobre critérios de morte encefálica e a escassez de doadores efetivos, limitam o alcance desse processo, evidenciando a necessidade de estratégias para ampliar a conscientização pública^(3–5)^.

Nesse contexto, a mídia, por meio das novelas, séries, filmes, campanhas publicitárias, revistas e jornais digitais, desempenha um papel de destaque como ferramenta informacional e promocional em questões relacionadas à saúde, visto que é um meio de fácil acesso e baixo custo para a obtenção de referências. A mídia, com isso, torna-se detentora do conhecimento e pode impactar, direta ou indiretamente, conforme o conteúdo e estruturação de suas publicações, as atitudes e tomada de decisão da comunidade, influenciando de forma positiva ou negativa o processo de doação de órgãos e tecidos^(6–9)^. Contudo, o impacto da mídia é ambivalente: conteúdos sensacionalistas ou deturpados podem reforçar mitos, prejudicando a confiança da sociedade^(8)^.

As publicações acerca da doação de órgãos de famosos, como o caso do apresentador brasileiro Augusto Liberato, que doou seus órgãos e tecidos após um acidente doméstico que causou sua morte encefálica em novembro de 2019 na Flórida, Estados Unidos da América, repercutiram em larga escala, sendo associadas ao aumento do número de doações pelo Registro Brasileiro de Transplantes^(10)^. Em contrapartida, notícias com informações deturpadas e apelativas sobre o tráfico de órgãos e diagnósticos errôneos desfavorecem a importância da doação de órgãos e tecidos^(8)^.

Apesar de a mídia executar um papel importante nas questões de saúde, a maior parte dos estudos disponíveis avalia as repercussões das mídias em campanhas publicitárias sobre tabagismo, alcoolismo, câncer, HIV/AIDS, entre outros^(6,9)^. Existe uma escassez de pesquisas nacionais que analisem especificamente o papel das publicações midiáticas no processo de doação de órgãos e tecidos. A maior parte dos trabalhos se limita a dados institucionais, como os da Associação Brasileira de Transplante de Órgãos e Ministério da Saúde, que são pouco acessíveis ao público geral. Essa lacuna torna relevante investigar como os veículos de maior alcance populacional — como jornais digitais — abordam o tema, já que são fontes primárias de informação para a maioria da população.

Diante disso, este estudo propõe-se a analisar a frequência e o conteúdo das publicações sobre doação e transplante de órgãos em dois jornais digitais de grande circulação no Brasil entre 2018 e 2023.

## MÉTODO

### Desenho do Estudo

Trata-se de estudo quanti-qualitativo de caráter documental acerca do conteúdo das reportagens e notícias veiculadas em dois jornais digitais de circulação nacional, sendo eles “O Estado de São Paulo”, conhecido como “Estadão”, e “Folha de São Paulo”.

### Local e População do Estudo

O estudo foi realizado em dois jornais digitais de grande circulação no Brasil. O jornal “O Estado de São Paulo” foi fundado, em 1875, com o nome “A Província de São Paulo”, quando o Brasil ainda vivia na Monarquia de Dom Pedro II. Em 1889, com a Proclamação da República, houve a mudança do nome para “O Estado de São Paulo”. O jornal apresenta-se na forma *online* desde 1995.

O jornal “Folha de São Paulo” surgiu em 1960 após a fusão de três diários, sendo eles “Folha da Manhã”, “Folha da Noite” e “Folha da Tarde”. Em 1995, é lançado o primeiro site de notícias *online*: a “FolhaWeb”.

### Critérios de Seleção

Foram incluídas todas as notícias que abordaram a temática na versão paga e gratuita. Houve a exclusão de quatro notícias, pois abordavam a doação de cadáveres e partes do corpo para fins científicos e questões pessoais que se sobrepunham à temática.

### Coleta de Dados

A coleta de dados ocorreu de setembro de 2023 a janeiro de 2024. Na primeira etapa, foi realizada a coleta das notícias e reportagens utilizando filtros com palavras-chave (doação de órgãos; doação de órgãos e tecidos; doação de tecidos; doador de órgãos; doador de tecidos; doador(es) vivo(s); doador(es) falecido(s); transplante de órgãos; transplante de órgãos e tecidos; transplante de tecidos; transplantado; transplantado de órgãos; transplantado de tecidos; tráfico de órgãos; recusa familiar da doação; recusa familiar; morte encefálica; morte encefálica; e doação) publicados no período de janeiro de 2018 a dezembro de 2023. Nessa fase, os artigos foram selecionados com base em seu título e resumo.

A segunda etapa compreendeu a leitura minuciosa das notícias e sua organização nos programas *Microsoft Excel* ^®^ e *Microsoft Word* ^®^ para aprofundar e ampliar a visualização dos conteúdos das publicações.

### Análise e Tratamento dos Dados

Na análise quantitativa, os dados foram extraídos da planilha do *Microsoft Excel* ^®^ e importados para o *software Statistical Package for the Social Sciences*, versão 20.0, para análise das frequências simples (n) e relativas (%). Para isso, os conteúdos das publicações foram agrupados de acordo com sua semelhança de significados no *Microsoft Excel* ^®^ e, posteriormente, enquadrados em categorias, sendo guiados pelo referencial de análise de conteúdo temática-categorial proposta por Bardin, que compreende as fases de pré-análise, exploração do material, e tratamento dos dados, inferência e interpretação^(11)^.

Para conceituar a categoria mídia, utilizou-se a mídia positiva para as notícias que promoviam e enalteciam a temática; negativa, para as notícias que depreciavam, problematizam e criticavam a temática^(12)^; e neutra, para aquelas que não expressaram um viés preponderante. A definição de cada código para as categorias foi realizada com leitura flutuante, conforme referencial. Após, definia-se o código conforme o conteúdo da notícia, e na sequência, foram realizadas avaliações por pares para confirmação das codificações para construção de cada categoria/classe, sendo as divergências dirimidas em rodadas de consenso em grupo.

A partir da análise, emergiram as codificações que geraram as categorias: mídia (positiva, negativa ou neutra); tema da notícia (doação, transplante ou transplante/doação); órgãos/tecidos e classe da notícia (incentivo/promoção, informação, impacto da pandemia de COVID-19, inovação, xenotransplante, desinformação acerca da doação/transplante, legislação, lista de espera, complicações no processo de doação/transplante, quebra de sigilo da identidade do doador, recusa familiar, e transporte de órgãos e tecidos).

Além disso, na análise qualitativa, foram utilizados os *softwares Interface de R pour les Analyses Multidimensionnelles de Textes et de Questionnaires* (IRAMUTEQ), versão 0.7 alpha 2, e R, versão 3.2.3. Dessa forma, os conteúdos das notícias e reportagens (*corpus* textual) foram transcritos para o *Microsoft Word* ^®^ e revisados para se adequar às especificações do *software*, como a introdução de linhas de comando, retirada de parágrafos, correção de pontuações, padronização das siglas e agrupamento das palavras significativas por *underline*. Para facilitar a análise, o IRAMUTEQ divide os textos do *corpus* em segmento de texto (ST), geralmente de três linhas^(13)^.

Para a presente pesquisa, utilizaram-se: 1) Nuvem de palavras, representação visual da frequência dos termos no *corpus* de acordo com seu tamanho; 2) Árvore de similitude, que é baseada na teoria de grafos, que indica as conexões entre as palavras; e 3) Dendrograma e apresentação fatorial, confeccionados através da Classificação Hierárquica Descendente (CHD), que agrupa as palavras em classes com o intuito de demonstrar a ligação delas entre si por meio do teste qui-quadrado (*χ* ^2^).

A interpretação das classes resultantes da CHD seguiu critérios teóricos fundamentados na análise de conteúdo^(11)^, combinada com a perspectiva da análise textual discursiva^(14)^. Cada classe foi examinada a partir dos vocábulos mais característicos (com maior valor de qui-quadrado), do conteúdo dos STs associados e da sua relação com o *corpus* completo. A atribuição de sentido às classes considerou tanto a frequência e associação dos termos quanto os contextos semânticos emergentes, possibilitando uma interpretação que extrapola a coocorrência lexical, ancorada na literatura da área temática da pesquisa. Essa abordagem parte de pressupostos epistemológicos construtivistas, compreendendo que o sentido dos discursos é construído socialmente e pode ser acessado por meio de estruturas lexicais compartilhadas. A análise textual clássica (CHD) utilizada pelo IRAMUTEQ busca identificar regularidades e padrões nos discursos, permitindo ao pesquisador interpretar significados latentes, preservando a coerência com os objetivos exploratórios e qualitativos da pesquisa^(15)^.

### Aspectos Éticos

Os dados coletados nesta pesquisa foram retirados de fontes documentais de domínio público. Conforme a Resolução nº 510/2016 do Conselho Nacional de Saúde, não há necessidade de avaliação do projeto pelo Comitê de Ética em Pesquisa.

## RESULTADOS

O banco de dados foi composto por 317 notícias e reportagens, sendo 185 (58,36%) publicações da “Folha de São Paulo” e 132 (41,64%) do “O Estado de São Paulo”. As publicações ocorreram no período de 2018 a 2023, tendo um número significativo no ano de 2023, com 159 notícias (50,16%), enquanto há um menor número no ano de 2020, com apenas 20 reportagens (6,31%). Ao examinar a distribuição das notícias em relação aos meses de suas publicações, agosto e setembro se mostram sobressalentes aos outros meses. Em relação ao conteúdo das notícias, 228 (71,9%) são consideradas positivas e 54 (17,03%), negativas. Já as temáticas estão associadas, sobretudo, ao transplante, com 192 (60,57%), seguido de doação, com 81 (25,55%) das publicações (Tabela 1).

**Tabela 1:** Caracterização das notícias publicadas nos jornais “O Estado de São Paulo” e “Folha de São Paulo” (2018–2023) – São Paulo, SP, Brasil, 2024.

Variáveis	N (%)
**Jornal**	
Folha de São Paulo	185 (58,36)
O Estado de São Paulo	132 (41,64)
**Mês**	
Janeiro	16 (5,05)
Fevereiro	8 (2,52)
Março	19 (5,99)
Abril	9 (2,84)
Maio	16 (5,05)
Junho	14 (4,42)
Julho	16 (5,05)
Agosto	85 (26,81)
Setembro	60 (18,93)
Outubro	25 (7,89)
Novembro	31 (9,78)
Dezembro	18 (5,68)
**Ano**	
2018	34 (10,73)
2019	26 (8,20)
2020	20 (6,31)
2021	34 (10,73)
2022	44 (13,88)
2023	159 (50,16)
**Mídia**	
Negativa	54 (17,03)
Neutra	35 (11,04)
Positiva	228 (71,92)
**Tema**	
Transplante	192 (60,57)
Doação	81 (25,55)
Doação/transplante	44 (13,88)

Para aprimorar a caracterização e quantificação das notícias, elas foram divididas em 12 classes, na qual a classe incentivo/promoção se mostrou predominante, com 107 (33,75%) das publicações, e, em último lugar, com apenas quatro notícias (1,27%), a classe transporte. Além disso, em 145 notícias (45,74%), não foram citados órgãos e/ou tecidos especificamente, seguidos do coração, com 95 notícias (29,97%) (Tabela 2).

**Tabela 2:** Classificação das notícias publicadas nos jornais “O Estado de São Paulo” e “Folha de São Paulo” quanto ao conteúdo e órgãos e tecidos doados/transplantados e citados – São Paulo, SP, Brasil, 2024.

Variáveis	N (%)
**Órgãos/tecidos detalhados**	
Não especificado	145 (45,74)
Coração	95 (29,97)
Rins	37 (11,67)
Fígado	12 (3,79)
Pulmão	8 (2,52)
Pâncreas	7 (2,21)
Pele	4 (1,26)
Útero	4 (1,26)
Intestino	2 (0,63)
Córneas	2 (0,63)
Ossos	1 (0,32)
**Classes**	
Incentivo/promoção	107 (33,75)
Informação	65 (20,51)
Impacto da pandemia de COVID-19	28 (8,83)
Inovação	28 (8,83)
Xenotransplante	25 (7,89)
Desinformação acerca da doação/transplante	15 (4,73)
Legislação	12 (3,79)
Lista de espera	10 (3,15)
Complicações no processo de doação/transplante	8 (3,52)
Quebra de sigilo da identidade do doador	8 (3,52)
Recusa familiar	7 (2,21)
Transporte de órgãos e tecidos	4 (1,27)

Na análise qualitativa, as palavras “paciente”, “ano”, “transplante”, “dizer”, “órgão”, “médico”, “coração”, entre outras, aparecem em destaque na nuvem de palavras (Figura 1). Em contrapartida, outras palavras se apresentam menos frequentes, como “receber”, “cirurgia”, “doador” e “procedimento”.

**Figura 1:** Nuvem de palavras identificadas nas reportagens dos jornais “O Estado de São Paulo” e “Folha de São Paulo” sobre doação e transplante de órgãos e tecidos – São Paulo, SP, Brasil, 2024.

Pode-se relacionar à árvore de similitude (Figura 2), que mostra a relação do ST “paciente” com a “fila de espera” de indivíduos (“pessoas”) que aguardam (“receber”) por um órgão ou tecido para transplante. A figura do “médico” faz referência aos profissionais que fazem parte da equipe que atua no processo de doação (“doador”) e (“transplante”) de órgãos e tecidos, e o “coração”.

**Figura 2:** Árvore de similitude das palavras encontradas nos textos publicados nos jornais “O Estado de São Paulo” e “Folha de São Paulo” referentes à doação e transplantes de órgãos e tecidos – São Paulo, SP, Brasil, 2024.

Na apresentação fatorial e CHD (Figura 3), o conteúdo gerou seis classes, com 20,4%, 12,8%, 10,5%, 21,5%, 22,3% e 12,5% dos STs, respectivamente. A classe 1 aborda a importância do indivíduo dizer para a família a sua vontade sobre a doação e a decisão que os familiares tomaram a partir disso; a classe 2 destaca as complicações de saúde que são utilizadas como critérios de prioridade para um transplante de órgãos e tecidos; a classe 3 fala sobre a importância da família decidir respeitar o desejo de doar os órgãos e tecidos; a classe 4 se refere aos estudos para utilizar os órgãos de porcos nos humanos (xenotransplante) como recurso para diminuir a escassez de órgãos para transplante e, com isso, diminuir a lista de espera; a classe 5 relaciona os impactos da COVID-19 às taxas de transplante no Brasil; e a classe 6 refere-se a questões relacionadas à divulgação dos casos nas redes sociais, como o transplante de coração do apresentador Faustão e a doação dos órgãos do apresentador Gugu.

**Figura 3:** Apresentação fatorial e dendrograma da Classificação Hierárquica Descendente, encontrados nos textos publicados nos jornais “O Estado de São Paulo” e “Folha de São Paulo”, referentes à doação e transplantes de órgãos e tecidos – São Paulo, SP, Brasil, 2024.

## DISCUSSÃO

A análise evidenciou o protagonismo dos meios digitais na promoção da doação e transplante de órgãos, especialmente por meio de conteúdos com viés positivo e caráter testemunhal capazes de mobilizar afetos e ampliar o debate público sobre o tema. A literatura corrobora esse achado ao demonstrar que narrativas transformacionais tendem a ser mais eficazes do que as exclusivamente informativas para gerar engajamento social^(6,16,17)^. Esse efeito é potencializado por estratégias indiretas, como a intensificação das publicações em datas comemorativas e o predomínio de tonalidades positivas nas matérias, o que contribui para uma percepção mais acolhedora do processo de doação^(6,16)^. Estudo publicado em 2023 analisou o conteúdo de 313 publicações em inglês acerca da doação de órgãos na plataforma de rede social TikTok^®^ e verificou que uma quantidade significativa dos vídeos promovia a doação de órgãos e combate à desinformação, com uma predominância de conteúdos positivos^(12)^. Dessa forma, pode-se afirmar que a disponibilidade de órgãos para transplante é influenciada pelo juízo social, que pode se modificar com estímulos adequados^(7)^.

Casos de grande repercussão, como os dos apresentadores brasileiros Fausto Silva (Faustão) e Augusto Liberato (Gugu), funcionam como gatilhos para a intensificação das publicações. Embora sirvam como oportunidade para esclarecer o funcionamento do sistema de transplantes e combater desinformações, essas coberturas também revelam a influência de fatores externos como a fama e o *status* social na visibilidade midiática do tema, o que gera percepções ambíguas sobre justiça e acesso ao sistema^(18)^. A resposta da imprensa diante dessas situações demonstra um potencial educativo importante, mas reforça a necessidade de diretrizes mais claras sobre a abordagem responsável dessas narrativas.

Diante disso, a predominância do tema “transplante” em relação à “doação” e a sub-representação de órgãos e tecidos específicos, como córneas, ossos e pele, revelam uma assimetria na cobertura jornalística. Tal desequilíbrio pode comprometer a compreensão pública sobre a interdependência entre essas etapas, conforme já apontado em outras investigações^(5,19,20)^. Ao priorizar o receptor e sua recuperação — frequentemente associado a sentimentos de superação e gratidão — em detrimento do processo de doação e da figura do doador, os meios de comunicação contribuem para uma narrativa parcial, que silencia a complexidade e a carga emocional envolvida na decisão familiar pela doação^(21)^.

Outro ponto crítico é o tratamento fragmentado das etapas do processo, que pode decorrer da estrutura editorial dos meios de comunicação. Embora compreensível do ponto de vista jornalístico, essa abordagem corre o risco de dificultar a percepção da doação e do transplante como um ciclo integrado, comprometendo o engajamento informado da população. Essa fragmentação também contribui para a invisibilidade de temas relevantes, como recusa familiar, morte encefálica e doação de tecidos — questões que exigem não apenas informação técnica, mas também sensibilidade cultural e ética^(4,5,22,23)^.

A pandemia de COVID-19 reforçou esse cenário de instabilidade ao impactar negativamente as taxas de doação e transplante por motivos clínicos, logísticos e de biossegurança^(24,25)^. Ainda assim, o uso ampliado da telemedicina para o acompanhamento de receptores demonstra a capacidade adaptativa do sistema, sugerindo possibilidades de inovação para o futuro.

Nesse contexto, destaca-se também o avanço das pesquisas sobre xenotransplante como uma resposta à escassez de doadores, com iniciativas nacionais e internacionais que reforçam a urgência de soluções complementares sem, no entanto, substituir a importância da doação humana^(26–28)^.

As evidências desta investigação indicam que o fortalecimento da cultura da doação requer um esforço articulado entre imprensa, profissionais de saúde e gestores públicos. A criação de guias orientativos, como o desenvolvido por instituições internacionais^(18)^, pode inspirar iniciativas nacionais voltadas à qualificação das práticas comunicacionais, combatendo o sensacionalismo e promovendo informação ética, clara e baseada em evidências. Esses achados apontam para a necessidade de articulação intersetorial entre mídia, serviços de saúde e instituições públicas, com vistas à construção de estratégias comunicacionais contínuas e responsáveis sobre a doação e o transplante de órgãos e tecidos. Recomenda-se o desenvolvimento de materiais de apoio — como guias para jornalistas — que contemplem aspectos éticos, técnicos e culturais, promovendo uma cobertura sensível, educativa e baseada em evidências.

Este estudo apresenta como limitação a análise restrita a dois jornais digitais de circulação nacional, o que pode não representar a diversidade de abordagens adotadas por veículos regionais, alternativos ou outras plataformas digitais. Além disso, o recorte temporal e o uso de palavras-chave específicas podem ter excluído conteúdos relevantes à temática. Por fim, ao privilegiar a análise do discurso publicado, não foi possível captar os efeitos da recepção dessas mensagens pelo público, o que limita inferências sobre seu impacto social direto.

Do ponto de vista da pesquisa, futuras investigações podem ampliar a análise para outros veículos midiáticos e redes sociais, bem como explorar a recepção das mensagens por diferentes públicos. Estudos qualitativos com familiares de potenciais doadores também podem aprofundar a compreensão sobre como as mensagens midiáticas impactam a decisão pela doação. Ademais, recomenda-se examinar as estratégias de comunicação sobre a doação de tecidos, cuja visibilidade ainda é limitada.

## CONCLUSÃO

Devido ao exposto, é possível evidenciar que, embora os jornais digitais analisem a temática da doação e transplante de órgãos e tecidos sob uma perspectiva positiva, a abordagem ainda é fragmentada, com ênfase no transplante em relação à doação. Esse desequilíbrio pode limitar a compreensão pública sobre a interdependência entre essas etapas e comprometer o engajamento da população com a causa.

Os resultados demonstram que eventos de grande repercussão midiática, como os casos envolvendo figuras públicas, influenciam diretamente o volume e o tom das publicações, indicando que a mídia responde a fatores temporários mais do que adota uma estratégia informacional contínua. Dessa forma, reforça-se a necessidade de uma articulação mais sólida entre profissionais de saúde, jornalistas e gestores públicos para promover uma cobertura ética e educativa, com o intuito de reduzir a desinformação e combater os estigmas. Investir em estratégias comunicativas é fundamental para fortalecer a cultura da doação no Brasil e reduzir a distância entre a informação divulgada e a tomada de decisão pela sociedade.

## Data Availability

Os dados da pesquisa só estão disponíveis mediante solicitação.

# Translation: Narrativas mediáticas sobre la donación y el trasplante de órganos y tejidos: análisis reflexivo del contenido publicado

**DOI:** 10.1590/1980-220X-REEUSP-2024-0342es

**Authors:** Gleyciane Santos Vieira, Rafael Rodrigo da Silva Pimentel, Rosane Almeida de Freitas, Marcelo José dos Santos

**Affiliations:** 1 Universidade de São Paulo, Escola de Enfermagem, São Paulo, SP, Brasil.; 2 Hospital Israelita Albert Einstein, Centro de Estudos, Pesquisa e Prática em Atenção Primária à Saúde e Redes, São Paulo, SP, Brasil.; 3 Universidade Estadual de Maringá, Maringá, PR, Brasil.

**Keywords:** Publicaciones Electrónicas, Obtención de Tejidos y Órganos, Noticias

## Abstract

**Objetivo:**

Analizar la frecuencia y el contenido de las publicaciones sobre donación y trasplante de órganos y tejidos en los periódicos digitales “Folha de São Paulo” y “O Estado de São Paulo” entre 2018 y 2023.

**Método:**

Mediante un enfoque cuantitativo-cualitativo, se examinaron 317 informes con base en criterios temáticos y discursivos, con la ayuda del software SPSS^®^ e IRAMUTEQ^®^.

**Resultados:**

Los datos revelaron un predominio de contenido positivo (71,9%), centrado en el tema del “trasplante” (60,57%), con picos en agosto y septiembre de 2023. Las categorías principales fueron “incentivo/promoción” e “información”, siendo el corazón el órgano más citado. El análisis textual destacó temas como el trasplante, la negativa familiar, el xenotrasplante y el impacto de la pandemia.

**Conclusión:**

Los medios digitales influyen significativamente en la percepción pública sobre la donación y el trasplante. Sin embargo, se hace hincapié en ciertas etapas del proceso, lo que puede comprometer su comprensión general. Las estrategias de comunicación continuas e integradas son esenciales para fortalecer la cultura de la donación.

## INTRODUCCIÓN

La donación de órganos y tejidos representa un acto de relevancia social capaz de salvar o mejorar la calidad de vida al ofrecer esperanza a miles de pacientes en lista de espera^(1)^. En Brasil, la demanda de trasplantes sigue creciendo, con cerca de 59 000 personas en espera de un órgano o tejido en 2023^(2)^. A pesar de los avances, los retos persistentes, como el rechazo familiar, la falta de conocimiento sobre los criterios de muerte cerebral y la escasez de donantes efectivos, limitan el alcance de este proceso, lo que pone de manifiesto la necesidad de estrategias para ampliar la concienciación pública^(3–5)^.

En este contexto, los medios de comunicación, a través de telenovelas, series, películas, campañas publicitarias, revistas y periódicos digitales, desempeñan un papel destacado como herramienta informativa y promocional en cuestiones relacionadas con la salud, ya que son un medio de fácil acceso y bajo costo para obtener referencias. De este modo, los medios de comunicación se convierten en depositarios del conocimiento y pueden influir, directa o indirectamente, según el contenido y la estructura de sus publicaciones, en las actitudes y la toma de decisiones de la comunidad, influyendo de forma positiva o negativa en el proceso de donación de órganos y tejidos^(6–9)^. Sin embargo, el impacto de los medios de comunicación es ambivalente: los contenidos sensacionalistas o tergiversados pueden reforzar los mitos, lo que perjudica la confianza de la sociedad^(8)^.

Las publicaciones sobre la donación de órganos de personas famosas, como el caso del presentador brasileño Augusto Liberato, que donó sus órganos y tejidos tras un accidente doméstico que le causó la muerte cerebral en noviembre de 2019 en Florida, Estados Unidos, tuvieron una gran repercusión y se asociaron con el aumento del número de donaciones por parte del Registro Brasileño de Trasplantes^(10)^. Por el contrario, las noticias con información distorsionada y sensacionalista sobre el tráfico de órganos y los diagnósticos erróneos perjudican la importancia de la donación de órganos y tejidos^(8)^.

Aunque los medios de comunicación desempeñan un papel importante en las cuestiones de salud, la mayoría de los estudios disponibles evalúan las repercusiones de los medios en las campañas publicitarias sobre el tabaquismo, el alcoholismo, el cáncer, el VIH/SIDA, entre otros^(6,9)^. Existe una escasez de investigaciones nacionales que analicen específicamente el papel de las publicaciones mediáticas en el proceso de donación de órganos y tejidos. La mayoría de los trabajos se limitan a datos institucionales, como los de la Asociación Brasileña de Trasplante de Órganos y el Ministerio de Salud, que son poco accesibles para el público en general. Esta laguna hace que sea relevante investigar cómo los medios de comunicación de mayor alcance poblacional, como los periódicos digitales, abordan el tema, ya que son fuentes primarias de información para la mayoría de la población.

En vista de ello, este estudio se propone analizar la frecuencia y el contenido de las publicaciones sobre donación y trasplante de órganos en dos periódicos digitales de gran circulación en Brasil entre 2018 y 2023.

## MÉTODO

### Diseño del Estudio

Se trata de un estudio cuantitativo-cualitativo de carácter documental sobre el contenido de los reportajes y noticias publicados en dos periódicos digitales de circulación nacional, a saber, “O Estado de São Paulo”, conocido como “Estadão”, y “Folha de São Paulo”.

### Lugar y Población del Estudio

El estudio se llevó a cabo en dos periódicos digitales de gran circulación en Brasil. El periódico “O Estado de São Paulo” fue fundado en 1875 con el nombre de “A Província de São Paulo”, cuando Brasil aún vivía bajo la monarquía de Dom Pedro II. En 1889, con la Proclamación de la República, se cambió el nombre por “O Estado de São Paulo”. El periódico se presenta en formato en línea desde 1995.

El periódico “Folha de São Paulo” surgió en 1960 tras la fusión de tres diarios: “Folha da Manhã”, “Folha da Noite” y “Folha da Tarde”. En 1995 se lanzó el primer sitio web de noticias en línea: “FolhaWeb”.

### Criterios de Selección

Se incluyeron todas las noticias que abordaban el tema en la versión de pago y en la gratuita. Se excluyeron cuatro noticias, ya que abordaban la donación de cadáveres y partes del cuerpo con fines científicos y cuestiones personales que se superponían al tema.

### Recolección de Datos

La recolección de datos se llevó a cabo entre septiembre de 2023 y enero de 2024. En la primera etapa, se realizó la recolección de noticias y reportajes utilizando filtros con palabras clave (donación de órganos; donación de órganos y tejidos; donación de tejidos; donante de órganos; donante de tejidos; donante(s) vivo(s); donante(s) fallecido(s); trasplante de órganos; trasplante de órganos y tejidos; trasplante de tejidos; trasplantado; trasplantado de órganos; trasplantado de tejidos; tráfico de órganos; rechazo familiar de la donación; rechazo familiar; muerte cerebral; muerte cerebral; y donación) publicados entre enero de 2018 y diciembre de 2023. En esta fase, los artículos se seleccionaron en función de su título y resumen.

La segunda etapa consistió en la lectura minuciosa de las noticias y su organización en los programas Microsoft Excel y Microsoft Word para profundizar y ampliar la visualización de los contenidos de las publicaciones.

### Análisis y Tratamiento de los Datos

En el análisis cuantitativo, los datos se extrajeron de la hoja de cálculo de *Microsoft Excel* ^®^ y se importaron al *software Statistical Package for the Social Sciences*, versión 20.0, para analizar las frecuencias simples (n) y relativas (%). Para ello, los contenidos de las publicaciones se agruparon según su similitud de significados en *Microsoft Excel* ^®^ y, posteriormente, se clasificaron en categorías, guiándose por el marco de referencia de análisis de contenido temático-categorial propuesto por Bardin, que comprende las fases de preanálisis, exploración del material y tratamiento de los datos, inferencia e interpretación^(11)^.

Para conceptualizar la categoría de medios de comunicación, se utilizó el término “positivo” para las noticias que promovían y elogiaban el tema; “negativo”, para las noticias que menospreciaban, problematizaban y criticaban el tema^(12)^; y “neutro”, para aquellas que no expresaban un sesgo predominante. La definición de cada código para las categorías se realizó mediante lectura flotante, según el referencial. A continuación, se definió el código según el contenido de la noticia y, a continuación, se realizaron evaluaciones por pares para confirmar las codificaciones para la construcción de cada categoría/clase, resolviéndose las divergencias en rondas de consenso en grupo.

A partir del análisis, surgieron las codificaciones que generaron las categorías: medios de comunicación (positivos, negativos o neutros); tema de la noticia (donación, trasplante o trasplante/donación); órganos/tejidos y clase de noticia (incentivo/promoción, información, impacto de la pandemia de COVID-19, innovación, xenotransplante, desinformación sobre la donación/trasplante, legislación, lista de espera, complicaciones en el proceso de donación/trasplante, violación del secreto de la identidad del donante, rechazo familiar y transporte de órganos y tejidos).

Además, en el análisis cualitativo se utilizaron los programas *Interface de R pour les Analyses Multidimensionnelles de Textes et de Questionnaires* (IRAMUTEQ), versión 0.7 alpha 2, y R, versión 3.2.3. De esta forma, los contenidos de las noticias y reportajes (*corpus* textual) se transcribieron a *Microsoft Word* ^®^ y se revisaron para adaptarlos a las especificaciones del *software*, como la introducción de líneas de comando, la eliminación de párrafos, la corrección de puntuación, la estandarización de siglas y la agrupación de palabras significativas mediante *subrayado*. Para facilitar el análisis, el IRAMUTEQ divide los textos del corpus en segmentos de texto (ST), generalmente de tres líneas^(13)^.

Para la presente investigación, se utilizaron: 1) Nube de palabras, representación visual de la frecuencia de los términos en el corpus según su tamaño; 2) Árbol de similitud, basado en la teoría de grafos, que indica las conexiones entre las palabras; y 3) Dendrograma y presentación factorial, elaborados mediante la Clasificación Jerárquica Descendente (CJD), que agrupa las palabras en clases con el fin de demostrar su conexión entre sí mediante la prueba de chi-cuadrado (*χ* ^2^).

La interpretación de las clases resultantes de la CJD siguió criterios teóricos basados en el análisis de contenido^(11)^, combinado con la perspectiva del análisis textual discursivo^(14)^. Cada clase se examinó a partir de los términos más característicos (con mayor valor de Chi-cuadrado), del contenido de los ST asociados y de su relación con el corpus completo. La atribución de significado a las clases consideró tanto la frecuencia y la asociación de los términos como los contextos semánticos emergentes, lo que permitió una interpretación que va más allá de la coocurrencia léxica, basada en la literatura del área temática de la investigación. Este enfoque parte de supuestos epistemológicos constructivistas, entendiendo que el significado de los discursos se construye socialmente y puede accederse a él a través de estructuras léxicas compartidas. El análisis textual clásico (CJD) utilizado por el IRAMUTEQ busca identificar regularidades y patrones en los discursos, lo que permite al investigador interpretar significados latentes, preservando la coherencia con los objetivos exploratorios y cualitativos de la investigación^(15)^.

### Aspectos Éticos

Los datos recopilados en esta investigación se obtuvieron de fuentes documentales de dominio público. De conformidad con la Resolución n.° 510/2016 del Consejo Nacional de Salud, no es necesario que el Comité de Ética en Investigación evalúe el proyecto.

## RESULTADOS

La base de datos constaba de 317 noticias e informes, de los cuales 185 (58,36%) fueron publicados por “Folha de São Paulo” y 132 (41,64%) por “O Estado de São Paulo”. Las publicaciones tuvieron lugar entre 2018 y 2023, con un número significativo en 2023, con 159 noticias (50,16%), mientras que hubo menos en 2020, con sólo 20 informes (6,31%). Al examinar la distribución de las noticias en relación con los meses en que se publicaron, agosto y septiembre destacan sobre los demás meses. En cuanto al contenido de las noticias, 228 (71,9%) se consideran positivas y 54 (17,03%) negativas. Los temas se asocian principalmente al trasplante, con 192 (60,57%), seguido de la donación, con 81 (25,55%) de las publicaciones (Tabla 1).

**Tabla 1:** Caracterización de las noticias publicadas en los periódicos “O Estado de São Paulo” y “Folha de São Paulo” (2018–2023) – São Paulo, SP, Brasil, 2024.

Variables	N (%)
**Periódico**	
Folha de São Paulo	185 (58,36)
O Estado de São Paulo	132 (41,64)
**Mes**	
Enero	16 (5,05)
Febrero	8 (2,52)
Marzo	19 (5,99)
Abril	9 (2,84)
Mayo	16 (5,05)
Junio	14 (4,42)
Julio	16 (5,05)
Agosto	85 (26,81)
Septiembre	60 (18,93)
Octubre	25 (7,89)
Noviembre	31 (9,78)
Diciembre	18 (5,68)
**Año**	
2018	34 (10,73)
2019	26 (8,20)
2020	20 (6,31)
2021	34 (10,73)
2022	44 (13,88)
2023	159 (50,16)
**Medios de comunicación**	
Negativo	54 (17,03)
Neutral	35 (11,04)
Positivo	228 (71,92)
**Sujeto**	
Trasplante	192 (60,57)
Donación	81 (25,55)
Donación/trasplante	44 (13,88)

Para mejorar la caracterización y cuantificación de las noticias, se dividieron en 12 clases, en las que predominó la clase incentivo/promoción, con 107 (33,75%) de las publicaciones, y en último lugar, con sólo cuatro noticias (1,27%), la clase transporte. Además, en 145 noticias (45,74%) no se mencionaron específicamente órganos y/o tejidos, seguido del corazón, con 95 noticias (29,97%) (Tabla 2).

**Tabla 2:** Clasificación de las noticias publicadas en los periódicos “O Estado de São Paulo” y “Folha de São Paulo” en términos de contenido y órganos y tejidos donados/trasplantados y mencionados – São Paulo, SP, Brasil, 2024.

Variables	N (%)
**Órganos/tejidos detallados**	
No especificados	145 (45,74)
Corazón	95 (29,97)
Riñón	37 (11,67)
Hígado	12 (3,79)
Pulmones	8 (2,52)
Páncreas	7 (2,21)
Piel	4 (1,26)
Útero	4 (1,26)
Intestino	2 (0,63)
Córneas	2 (0,63)
Huesos	1 (0,32)
**Clases**	
Incentivo/promoción	107 (33,75)
Información	65 (20,51)
Impacto de la pandemia COVID-19	28 (8,83)
Innovación	28 (8,83)
Xenotrasplantes	25 (7,89)
Desinformación sobre donación/trasplante	15 (4,73)
Legislación	12 (3,79)
Lista de espera	10 (3,15)
Complicaciones en el proceso de donación/trasplante	8 (3,52)
Violación de la confidencialidad de la identidad del donante	8 (3,52)
Negativa de la familia	7 (2,21)
Transporte de órganos y tejidos	4 (1,27)

En el análisis cualitativo, las palabras “paciente”, “año”, “trasplante”, “decir”, “órgano”, “médico”, “corazón”, entre otras, aparecen destacadas en la nube de palabras (Figura 1). Por el contrario, otras palabras aparecen con menos frecuencia, como “recibir”, “cirugía”, “donante” y “procedimiento”.

**Figura 1:** Nube de palabras (en portugués) identificadas en los reportajes de los periódicos “O Estado de São Paulo” y “Folha de São Paulo” sobre la donación y el trasplante de órganos y tejidos – São Paulo, SP, Brasil, 2024.

Se puede relacionar con el árbol de similitudes (Figura 2), que muestra la relación del ST “paciente” con la “lista de espera” de individuos (“personas”) que esperan (“recibir”) un órgano o tejido para trasplante. La figura del “médico” hace referencia a los profesionales que forman parte del equipo que participa en el proceso de donación (“donante”) y (“trasplante”) de órganos y tejidos, y el “corazón”.

**Figura 2:** Árbol de similitud de las palabras en portugués encontradas en los textos publicados en los periódicos “O Estado de São Paulo” y “Folha de São Paulo” referidos a la donación y los trasplantes de órganos y tejidos – São Paulo, SP, Brasil, 2024.

En la presentación factorial y CJD (en español en Figura 3, derecha), el contenido generó seis clases, con 20,4%, 12,8%, 10,5%, 21,5%, 22,3% y 12,5% de los ST, respectivamente. La clase 1 aborda la importancia de que el individuo comunique a su familia su voluntad sobre la donación y la decisión que los familiares tomaron a partir de ello; la clase 2 destaca las complicaciones de salud que se utilizan como criterios de prioridad para un trasplante de órganos y tejidos; la clase 3 habla de la importancia de que la familia decida respetar el deseo de donar los órganos y tejidos; la clase 4 se refiere a los estudios para utilizar órganos de cerdos en humanos (xenotransplante) como recurso para reducir la escasez de órganos para trasplante y, con ello, reducir la lista de espera; la clase 5 relaciona los impactos de la COVID-19 con las tasas de trasplante en Brasil; y la clase 6 se refiere a cuestiones relacionadas con la divulgación de los casos en las redes sociales, como el trasplante de corazón del presentador Faustão y la donación de órganos del presentador Gugu.

**Figura 3:** Presentación factorial y dendrograma de la Clasificación Jerárquica Descendente, encontrados en los textos publicados en los periódicos “O Estado de São Paulo” y “Folha de São Paulo”, referentes a la donación y los trasplantes de órganos y tejidos – São Paulo, SP, Brasil, 2024.

## DISCUSIÓN

El análisis evidenció el protagonismo de los medios digitales en la promoción de la donación y el trasplante de órganos, especialmente a través de contenidos con un sesgo positivo y de carácter testimonial, capaces de movilizar afectos y ampliar el debate público sobre el tema. La literatura corrobora este hallazgo al demostrar que las narrativas transformacionales tienden a ser más eficaces que las exclusivamente informativas para generar compromiso social^(6,16,17)^. Este efecto se ve potenciado por estrategias indirectas, como la intensificación de las publicaciones en fechas conmemorativas y el predominio de tonos positivos en los artículos, lo que contribuye a una percepción más acogedora del proceso de donación^(6,16)^. Un estudio publicado en 2023 analizó el contenido de 313 publicaciones en inglés sobre la donación de órganos en la plataforma de redes sociales TikTok^®^ y verificó que una cantidad significativa de los videos promovía la donación de órganos y combatía la desinformación, con un predominio de contenidos positivos^(12)^. De este modo, se puede afirmar que la disponibilidad de órganos para trasplantes está influenciada por el juicio social, que puede modificarse con los estímulos adecuados^(7)^.

Los casos de gran repercusión, como los de los presentadores brasileños Fausto Silva (Faustão) y Augusto Liberato (Gugu), funcionan como desencadenantes para la intensificación de las publicaciones. Aunque sirven como oportunidad para aclarar el funcionamiento del sistema de trasplantes y combatir la desinformación, estas coberturas también revelan la influencia de factores externos como la fama y el estatus social en la visibilidad mediática del tema, lo que genera percepciones ambiguas sobre la justicia y el acceso al sistema^(18)^. La respuesta de la prensa ante estas situaciones demuestra un importante potencial educativo, pero refuerza la necesidad de directrices más claras sobre el enfoque responsable de estas narrativas.

Ante esto, el predominio del tema “trasplante” en relación con la “donación” y la infrarrepresentación de órganos y tejidos específicos, como córneas, huesos y piel, revelan una asimetría en la cobertura periodística. Este desequilibrio puede comprometer la comprensión pública sobre la interdependencia entre estas etapas, como ya se ha señalado en otras investigaciones^(5,19,20)^. Al priorizar al receptor y su recuperación —a menudo asociada a sentimientos de superación y gratitud— en detrimento del proceso de donación y de la figura del donante, los medios de comunicación contribuyen a una narrativa parcial, que silencia la complejidad y la carga emocional que implica la decisión familiar de donar^(21)^.

Otro punto crítico es el tratamiento fragmentado de las etapas del proceso, que puede derivarse de la estructura editorial de los medios de comunicación. Aunque comprensible desde el punto de vista periodístico, este enfoque corre el riesgo de dificultar la percepción de la donación y el trasplante como un ciclo integrado, lo que compromete el compromiso informado de la población. Esta fragmentación también contribuye a la invisibilidad de temas relevantes, como el rechazo familiar, la muerte cerebral y la donación de tejidos, cuestiones que requieren no solo información técnica, sino también sensibilidad cultural y ética^(4,5,22,23)^.

La pandemia de COVID-19 ha reforzado este escenario de inestabilidad al afectar negativamente a las tasas de donación y trasplante por motivos clínicos, logísticos y de bioseguridad^(24,25)^. Aun así, el uso ampliado de la telemedicina para el seguimiento de los receptores demuestra la capacidad de adaptación del sistema, lo que sugiere posibilidades de innovación para el futuro.

En este contexto, también cabe destacar el avance de las investigaciones sobre el xenotrasplante como respuesta a la escasez de donantes, con iniciativas nacionales e internacionales que refuerzan la urgencia de soluciones complementarias sin, sin embargo, sustituir la importancia de la donación humana^(26–28)^.

Las evidencias de esta investigación indican que el fortalecimiento de la cultura de la donación requiere un esfuerzo coordinado entre la prensa, los profesionales de la salud y los gestores públicos. La creación de guías orientativas, como la desarrollada por instituciones internacionales^(18)^, puede inspirar iniciativas nacionales destinadas a mejorar las prácticas comunicativas, combatiendo el sensacionalismo y promoviendo una información ética, clara y basada en la evidencia. Estos hallazgos apuntan a la necesidad de una articulación intersectorial entre los medios de comunicación, los servicios de salud y las instituciones públicas, con miras a la construcción de estrategias comunicativas continuas y responsables sobre la donación y el trasplante de órganos y tejidos. Se recomienda el desarrollo de materiales de apoyo, como guías para periodistas, que contemplen aspectos éticos, técnicos y culturales, promoviendo una cobertura sensible, educativa y basada en la evidencia.

Esta investigación presenta como limitación el análisis restringido a dos periódicos digitales de circulación nacional, lo que puede no representar la diversidad de enfoques adoptados por medios regionales, alternativos u otras plataformas digitales. Además, el periodo de tiempo y el uso de palabras clave específicas pueden haber excluido contenidos relevantes para el tema. Por último, al privilegiar el análisis del discurso publicado, no fue posible captar los efectos de la recepción de estos mensajes por parte del público, lo que limita las inferencias sobre su impacto social directo.

Desde el punto de vista de la investigación, futuras investigaciones pueden ampliar el análisis a otros medios de comunicación y redes sociales, así como explorar la recepción de los mensajes por diferentes públicos. Los estudios cualitativos con familiares de posibles donantes también pueden profundizar la comprensión sobre cómo los mensajes mediáticos impactan en la decisión de donar. Además, se recomienda examinar las estrategias de comunicación sobre la donación de tejidos, cuya visibilidad aún es limitada.

## CONCLUSIÓN

Por lo expuesto, se puede evidenciar que, aunque los periódicos digitales analizan el tema de la donación y el trasplante de órganos y tejidos desde una perspectiva positiva, el enfoque sigue siendo fragmentado, con énfasis en el trasplante en relación con la donación. Este desequilibrio puede limitar la comprensión pública sobre la interdependencia entre estas etapas y comprometer el compromiso de la población con la causa.

Los resultados demuestran que los eventos de gran repercusión mediática, como los casos que involucran a figuras públicas, influyen directamente en el volumen y el tono de las publicaciones, lo que indica que los medios de comunicación responden a factores temporales más que adoptar una estrategia informativa continua. De este modo, se refuerza la necesidad de una articulación más sólida entre los profesionales de la salud, los periodistas y los gestores públicos para promover una cobertura ética y educativa, con el fin de reducir la desinformación y combatir los estigmas. Invertir en estrategias comunicativas es fundamental para fortalecer la cultura de la donación en Brasil y reducir la distancia entre la información difundida y la toma de decisiones por parte de la sociedad.

## Data Availability

Los datos de la encuesta solo están disponibles previa solicitud.
